# Induced Systemic Resistance against *Botrytis cinerea* by *Bacillus cereus* AR156 through a JA/ET- and *NPR1*-Dependent Signaling Pathway and Activates PAMP-Triggered Immunity in *Arabidopsis*

**DOI:** 10.3389/fpls.2017.00238

**Published:** 2017-02-28

**Authors:** Pingping Nie, Xia Li, Shune Wang, Jianhua Guo, Hongwei Zhao, Dongdong Niu

**Affiliations:** ^1^Department of Plant Pathology, College of Plant Protection, Nanjing Agricultural UniversityNanjing, China; ^2^Key Laboratory of Integrated Management of Crop Diseases and Pests, Nanjing Agricultural University, Ministry of EducationNanjing, China

**Keywords:** *Bacillus cereus* AR156, induced systemic resistance, salicylic acid (SA), jasmonic acid (JA), ethylene (ET)

## Abstract

Induced resistance response is a potent and cost effective plant defense against pathogen attack. The effectiveness and underlying mechanisms of the suppressive ability by *Bacillus cereus* AR156 to *Pseudomonas syringae* pv. *tomato* DC3000 (*Pst* DC3000) in *Arabidopsis* has been investigated previously; however, the strength of induced systemic resistance (ISR) activity against *Botrytis cinerea* remains unknown. Here, we show that root-drench application of AR156 significantly reduces disease incidence through activation of ISR. This protection is accompanied with multilayered ISR defense response activated via enhanced accumulation of PR1 protein expression in a timely manner, hydrogen peroxide accumulation and callose deposition, which is significantly more intense in plants with both AR156 pretreatment and *B. cinerea* inoculation than that in plants with pathogen inoculation only. Moreover, AR156 can trigger ISR in *sid2-2* and *NahG* mutants, but not in *jar1, ein2* and *npr1* mutant plants. Our results indicate that AR156-induced ISR depends on JA/ET-signaling pathway and *NPR1*, but not SA. Also, AR156-treated plants are able to rapidly activate MAPK signaling and *FRK1*/*WRKY53* gene expression, both of which are involved in pathogen associated molecular pattern (PAMP)-triggered immunity (PTI). The results indicate that AR156 can induce ISR by the JA/ET-signaling pathways in an *NPR1*-dependent manner and involves multiple PTI components.

## Introduction

Plants are protected from pathogen attack through activation of innate immune system, which is a consequence of co-evolution between plants and their pathogens (Jones and Dangl, 2006). The emergence of pathogens is first detected by pattern recognition receptors (PRRs) that are localized on plant cell membrane. PRRs percept the conserved pathogen identification molecules known as pathogen-associated molecular patterns (PAMPs), which include flagellin, lipopolysaccharides, glycoproteins, or chitin (Jones and Dangl, 2006). The interaction between PRRs and PAMPs consequently triggers the so-called PAMP-triggered immunity (PTI), which initiates many immune responses, including oxidative burst, callose deposition, activation of the MAPK (mitogen-activated protein kinase) cascade, and defense-related gene expression (Altenbach and Robatzek, 2007; Schwessinger and Zipfel, 2008). During the course of evolution, some successful pathogens emerged that can successfully infect plants by suppressing PTI. This suppression is achieved by secreting virulent proteins generically termed effectors, which causes effector-triggered susceptibility (ETS) consequently (Speth et al., 2007). In response to effectors, some plants have evolved resistance (R) proteins, which can recognize effectors directly or indirectly, and elicit effector-triggered immunity (ETI). ETI usually is accompanied with a hypersensitive response (HR) at the infection site, which is thought to restrict biotrophic pathogen growth (Chisholm et al., 2006; Jones and Dangl, 2006).

Beyond defense response against an intermediate infection, resistance can be induced by a temporarily prior infection that is effective for a certain period of time, and to a broad spectrum of pathogens temporarily after (Fu and Dong, 2013). The induced defense responses can be activated by pathogen infection, microbial symbiosis, or other elicitation, such as wounding. There are two types of induced resistances that are phenotypically hard-to-distinguished: the induced systemic resistance (ISR) and systemic acquired resistance (SAR). ISR is a systemic resistance induced by some non-pathogenic rhizobacteria that can suppress disease in plants (van Loon et al., 1998). In contrast, SAR is an induced resistance that develops in whole plants in response to a temporally earlier local exposure to a pathogen. In both SAR and ISR, phytohormone signaling pathways, such as salicylic acid (SA), jasmonic acid (JA), and ethylene (ET) are found to play crucial regulatory roles (Glazebrook, 2001).

Induced systemic resistance (ISR) has been reported in many plant species, such as rice, bean, carnation, cucumber, radish, tobacco, tomato, and *Arabidopsis*, which is effective against a broad spectrum of plant pathogens, ranging from fungi, bacteria, to viruses, and even to insect herbivores (Van der Ent et al., 2008; Pieterse et al., 2014). ISR requires JA and ET signaling pathways and is associated with the expression of the gene encoding plant defensin 1.2 (*PDF1.2*) (Van Oosten et al., 2008). For example, the rhizobacterial strain *Pseudomonas fluorescens* WCS417r has been shown to trigger ISR in several plant species and in *Arabidopsis*, where it functions through JA/ET signaling pathways and in a *NPR1*-dependent manner (Pieterse et al., 2002). However, dependence on both SA- and JA/ET-signaling pathways is also observed. For example, we previously reported that the ISR mediated by the rhizobacterium *Bacillus cereus* strain AR156 requires both the SA and JA/ET signaling pathways and *NPR1* (Niu et al., 2011). Also, colonization of *Arabidopsis* roots by *Trichoderma atroviride* IMI 206040 induces the expression of SA and JA/ET pathways simultaneously to confer resistance against hemibiotrophic and necrotrophic phytopathogens (Salas-Marina et al., 2011).

In most cases, ISR is associated with a potentiated defensive capacity, which is termed “priming”. Priming does not cause a direct induction of resistance-related genes, or enhance the production of phytohormones or hormone-responsive genes in systemic tissues. Instead, ISR enhances the sensitivity to hormones rather than their synthesis. Therefore, priming is cost-effective in increasing plant resistance and is more efficient in activating defense mechanisms upon pathogen attack (Conrath et al., 2006; Pastor et al., 2013). Beneficial rhizobacteria trigger ISR by priming the plant for potentiated activation of varieties of cellular defense responses, such as oxidative burst (Ahn et al., 2007), cell-wall reinforcement (Heil and Bostock, 2002), defense-related enzymes accumulation (Rahman et al., 2014), and secondary metabolites production (Yedidia et al., 2003).

In our previous study, the beneficial bacterium *B*. *cereus* AR156 was demonstrated to trigger ISR in *Arabidopsis* through both the SA- and JA/ET- signaling pathways, which lead to enhanced resistance to bacterial infection (Niu et al., 2011). As an effort to further dissect the mechanism of AR156-mediated ISR, we are prompted to explore more protective potential of this bacterium. *Botrytis cinerea*, a necrotrophic fungus causing gray mold disease, is considered an important pathogen around the world. In this study, we show that *B. cereus* AR156 treatment inhibits *B. cinerea* infection in *Arabidopsis* through activation of ISR. The potency of induced protection is lost in *jar1, ein2* and *npr1* mutant but unaffected in *sid2-2* and *NahG* plants, implicating the JA/ET signaling pathway and *NPR1* are required but the SA signaling pathway is dispensable for AR156-induced ISR to *B. cinerea* infection. We also show that the primed defense in AR156-treated plants is mediated by enhanced activation of multiple PTI defense responses.

## Materials and methods

### Plants and growth conditions

*Arabidopsis thaliana* plants were maintained at 22°C with a 12-h light/12-h dark photoperiod. *Arabidopsis thaliana* ecotype Col-0 and mutants were cultivated in vermiculite. All plants were used for experiments when they were 4 weeks old. The mutants used in this study (*sid2-2, NahG, jar1, ein2, npr1*) were described elsewhere (Staswick et al., 1992; Bowling et al., 1994; Delaney, 1994; Alonso et al., 1999).

### AR156 treatment, pathogen inoculation, and disease assays

*Bacillus cereus* AR156 was grown on Luria-Bertani (LB) agar plates at 28°C for 24 h. Subsequently, bacterial cells were pelleted by centrifugation and were resuspended in sterile 0.85% NaCl with a final concentration of 5 × 10^8^ CFU/ml. For a protection assay, AR156 or corresponding mock (0.85% NaCl) was root-drench applied 7 days prior infection. For fungal infection experiments, 4-weeks-old plants were used. *Botrytis cinerea* strain B1301 was cultivated on PSA agar medium for 7 days. Spores were collected in *B. cinerea* infection buffer to prepare the inoculum and adjusted to a final concentration of 1 × 10^6^ spores/ml. Inoculation was carried out by depositing a 10 ul droplet on each side of the midvein. Ten inoculated plants for each genotype were placed in plant growth room maintained at a high humidity. For each time point, at least three biological replicates were analyzed. In planta fungal growth was examined by analyzing the transcript levels of *B. cinerea* actin gene (*BcActin*) using primer BcActin-1F (5′-TCC AAG CGT GGT ATT CTT ACC C-3′) and BcActin-1R (5′- TGG TGC TAC ACG AAG TTC GTT G-3′). The *Arabidopsis* actin gene (*AtActin2*) amplified by primer AtActin2-1F (5′-GGC GAT GAA GCT CAA TCC AAA CG-3′) and AtActin2-1R (5′-GGT CAC GAC CAG CAA GAT CAA GAC G-3′) was used as an internal control.

### RNA extraction and qRT-PCR analysis of gene expression

Total RNA was extracted from *Arabidopsis* leaves with TRIzol Reagent (Invitrogen, San Diego, CA, U.S.A). In brief, 1 ug total RNA was used for cDNA synthesis by using a commercial reverse transcription system (TaKaRa Biotech, Dalian, China). After the cDNA was diluted 10 times, 2 ul diluted cDNA was used for real-time quantitative PCR with the following program: 40 cycles at 95°C for 30 s, 55°C for 30 s, and 72°C for 34 s. Three replications were performed for each sample. The data were normalized with *AtActin*, and the means of three replications were presented. Primers used in qRT-PCR were listed in Table S1.

### Examination of hydrogen peroxide accumulation and callose deposition

Hydrogen peroxide accumulation and callose deposition examination was performed according to previously described procedures (Niu et al., 2011). Briefly, for accumulation of ROS, *Arabidopsis* leaves from at least three different plants were stained with DAB solution (1 mg of diaminobenzidine per milliliter, pH 3.8) for 8 h dark at 25 to 28°C. After being cleared with 96 % (vol/vol) ethanol and preserved in 50% (vol/vol) ethanol, hydrogen peroxide was visualized as dark-brown precipitate under the light microscope. For callose deposition, *Arabidopsis* leaves were immerged in 5 ml of destaining solution (Acetic acid/ethanol = 5:95) (vol/vol) and were infiltrated by applying a vacuum for 5 to 10 min. Leaves were incubated in a 60°C water bath for 20 to 30 min to clear chlorophyll. The chlorophyll-free leaves were gently rinsed with water and were then soaked in 3 to 5 ml of 0.01% (wt/vol) aniline blue staining solution containing 150 mM K_2_HPO_4_ (pH 9.5) kept in dark for 2 to 4 h. After staining, *Arabidopsis* leaves were gently rinsed with water and were then mounted on microscope slides that were observed under an epifluorescence microscope with a UV excitation filter. Levels of callose deposition were quantified using Imge J software and expressed relative to total leaf area as described (Luna et al., 2011).

### Protein extraction and analysis

Plant tissue was ground in liquid nitrogen and total proteins were extracted using 2 × SDS loading buffer. The samples were resolved on SDS–PAGE gels and transferred onto nitrocellulose membranes. The blots were probed with appropriate antibodies: monoclonal mouse anti-α tubulin (1:4,000 dilution); polyclonal rabbit anti-PR1 (1:2,000 dilution). For MAPK activity assay, sample were analyzed by Western blotting using monoclonal rabbit phospho-p44/42 MAPK (Erk1/2) (Thr202/Tyr204) (D13.14.4E) XP antibodies (Cell Signaling Technology, #4370S, 1:2,000 dilution). For these assays, α-tubulin was used as a loading control.

## Results

### *B. cereus* AR156 induces an effective ISR against *Botrytis cinerea* infection

**Figure 1:**
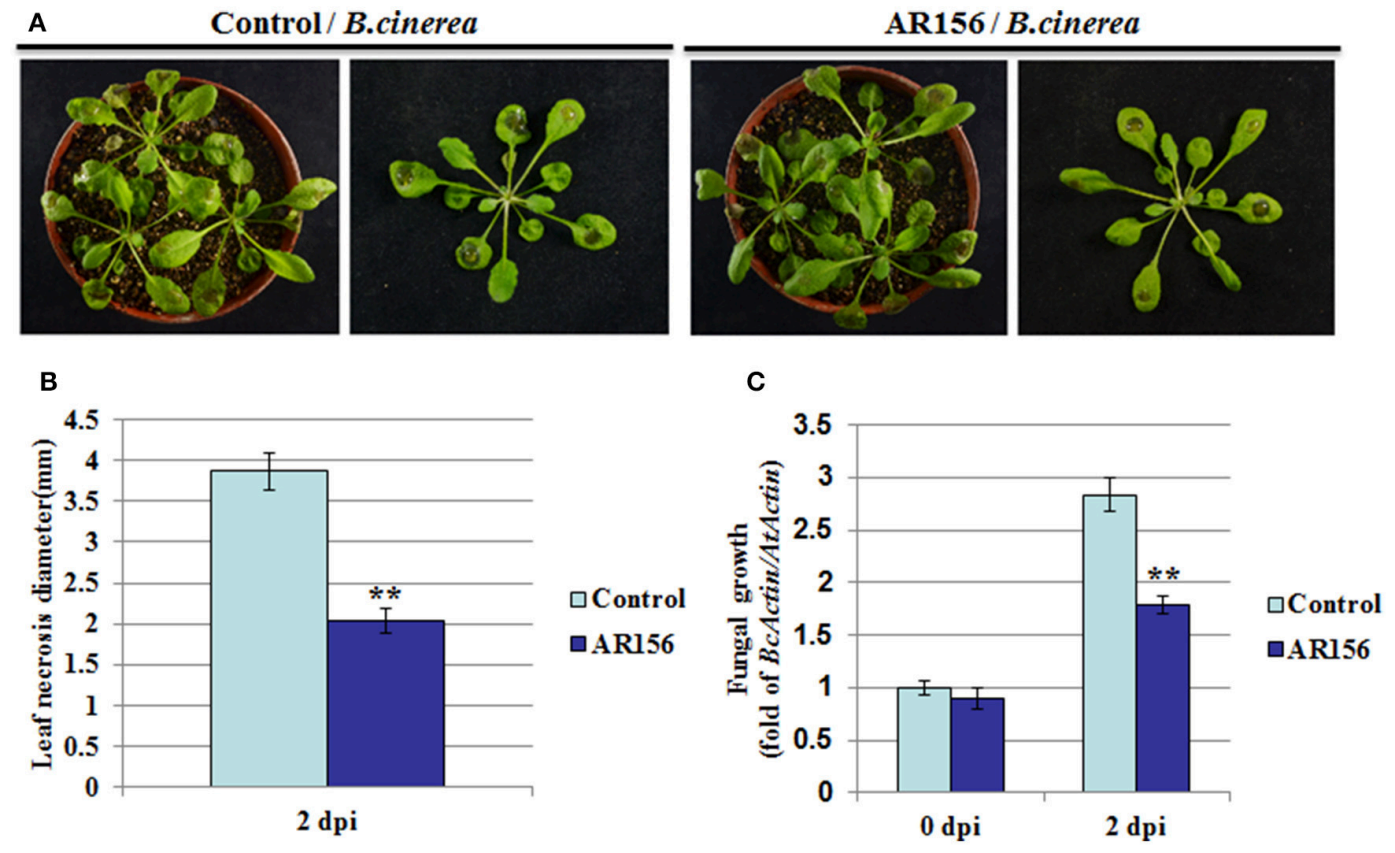
***B. cereus*** **AR156 induces an effective ISR against *B. cinerea* infection**. *Arabidopsis* Col-0 plants are drench-applied with AR156 at 5 × 10^8^ CFU/ml or 0.85% NaCl (Control). Control-or AR156-treated plants (7 days) are inoculated by depositing a 10 ul droplet of *B. cinerea* spores (1 × 10^6^ spores/ml) on each side of the midvein. **(A)** Disease symptoms observation and photo talking were made 2 days post infection (dpi). **(B)** Leaf necrosis development was evaluated at 2 dpi by measuring the average necrosis diameter on five leaves per plants for 10 plants (Col-0). **(C)** In planta growth of *B. cinerea*. Measurement of fungal growth was carried out by simultaneous quantification of the expression levels of *B. cinerea Actin* gene (*BcActin*) and the *Arabidopsis Actin* gene (*AtActin*). Relative fungal growth was determined by ratios of *BcActin/AtActin*. A Student's *t*-test was used to determine significant differences between the AR156-treated sample and the control (^**^P < 0.01). The means values ± *SD* (*n* = 12) from one representative experiment among three independent repeats are shown.

We previously found in *Arabidopsis* that *B. cereus* AR156 could prime the whole plant for an induced resistance to *Pst* DC3000 infection. In this study, we designed experiments to test whether AR156 is able to induce ISR against *B. cinerea* as well. *Arabidopsis* was first pretreated with AR156 or mock (0.85% NaCl) for 7 days in a root-drench application manner, followed by *B. cinerea* infection. Effect of AR156 on ISR was examined by the plant performance against pathogen infection. Two days after pathogen inoculation, mock-treated plants showed typical symptoms of *B. cinerea* disease-severe necrosis around inoculating loci, leaves yellowing, and water-soaked spots surrounded by the spores (Figure 1A). In contrast, plants with AR156-pretreatment exhibited a significant (*P* < 0.01) reduction on disease symptoms, manifested by smaller necrosis size, less yellowing, compared with mock-treated plants (Figure 1B). As an indicator of the effectiveness of elimination of pathogen infection, fungal hyphae were measured 2 days after inoculation (dpi) by qRT-PCR. In agreement with the reduced disease symptom observed on leaf surface, significantly reduced fungal hyphae were detected inside leaf tissue from AR156-pretreated plants than that with control pre-treatment, indicating AR156 effectively protected *Arabidopsis* from *B. cinerea* infection (Figure 1C).

### AR156 induces ISR by potentiating PR1 expression, hydrogen peroxide accumulation, and callose deposition in Arabidopsis

**Figure 2:**
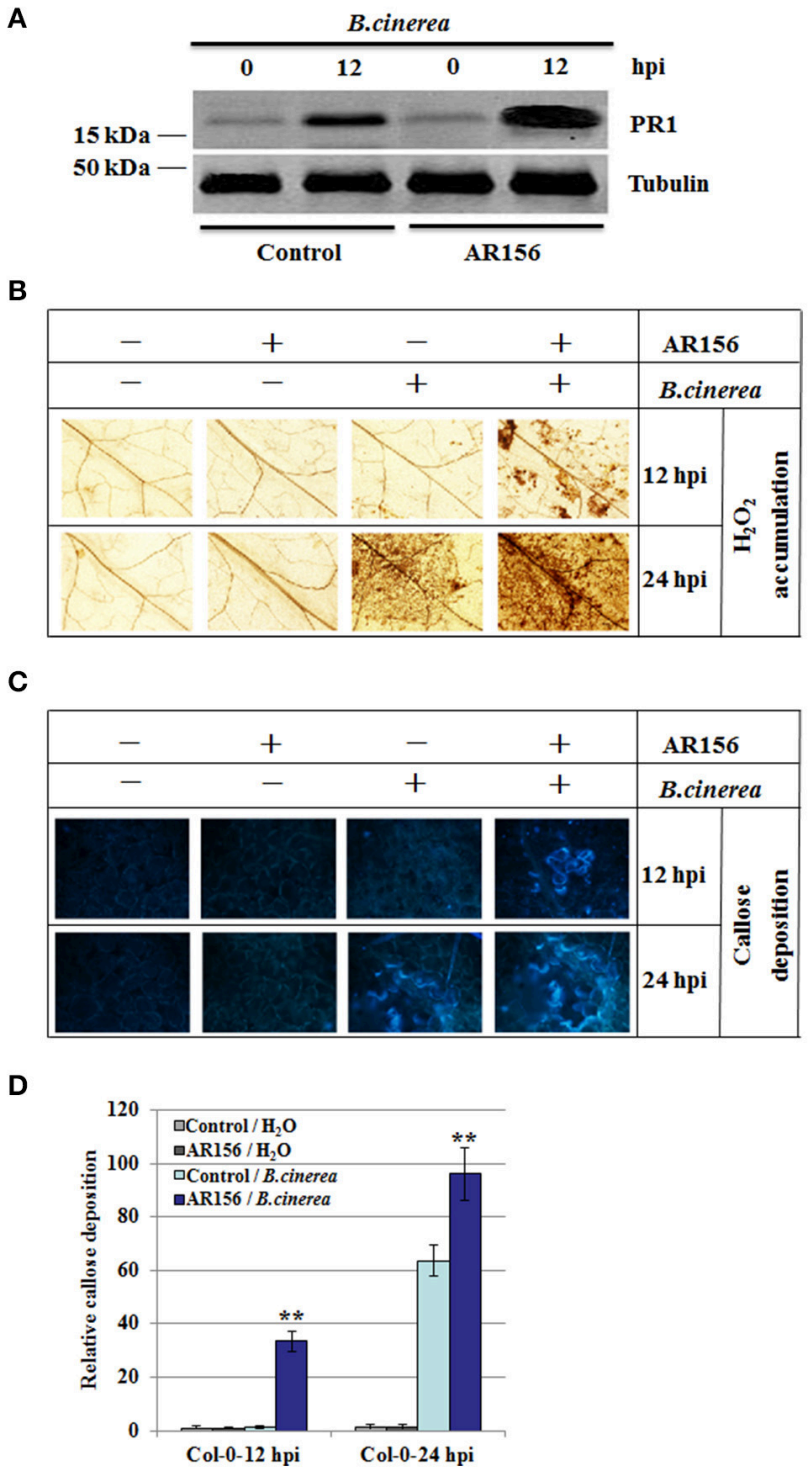
**AR156 pretreatment induces PR1 protein expression, H_2_O_2_ accumulation and callose deposition in systemic leaves infected with *B. cinerea Arabidopsis* Col-0 plants were infected by *B***. *cinerea* spore suspension (1 × 10^6^ spores/ml), and the leaves were collected at 12 hpi and 24 hpi, respectively. **(A)** PR1 was detected by an antibody specifically recognizes PR1; Tubulin was used as an equal loading control. **(B–D)** ROS accumulation **(B)** and callose deposition **(C)** were detected in plants with AR156- or control-pretreatment followed by *B. cinerea* or mock infection (Solution). ROS accumulation was detected by DAB staining; Callose deposition were observed under light and epifluorescence microscopes with a UV excitation filter. Relative callose quantities in droplet inoculated leaves of 4-week-old plants. Callose was quantified from digital microscopy photographs **(D)**. Shown are mean areas of callose per leaf relative to total leaf area ± *SD* (*n* = 24). A Student's *t*-test was used to determine significant differences between the AR156-treated sample and the control (^**^P < 0.01). Similar results were obtained in three independent repeats.

Establishment of ISR is usually accompanied with potentiated activation of various cellular defense responses against pathogen infection, which was called priming (Conrath et al., 2001). To investigate whether AR156-induced ISR was accompanied with primed defense responses in systemic leaves, we measured the expression level of defense-related protein PR1. With AR156 or control pre-treatment alone, PR1 expression was almost negligible prior pathogen infection, indicating AR156 by itself in not capable of inducing PR1 expression (Figure 2A). At 12 hpi, elevated PR1 expression was detected in *B. cinerea*-infected leaves, indicating the *Arabidopsis* innate immunity system recognizes and responds rapidly against *B. cinerea* infection. Moreover, plants with AR156 pre-treatment accumulated much higher PR1 than the control treated plants did. These results indicate that in plants with AR156 pretreatment, the innate immunity system is at a potentiated status so that plants could launch a much accelerated defense response upon infection (Figure 2A).

To investigate the molecular mechanism of AR156-mediated ISR, H_2_O_2_ accumulation and callose deposition pattern were examined in plants inoculated with *B. cinerea*, with or without AR156 pretreatment. H_2_O_2_ accumulation and callose deposition are two rapid responses elicited by pathogen infection, intensity and rapidity of which are hallmarks of successful immune response. AR156 neither induce the production of H_2_O_2_ at 12 hpi, nor 24 hpi, indicating that AR156 itself is not an elicitor of plant defense (Figure 2B). At 12 hpi, *B. cinerea* infection alone did not induce measurable H_2_O_2_ accumulation, indicating at this stage of infection, plants were not able to deploy an effective defense response yet, in term of H_2_O_2_ accumulation. However, in plants pretreated with AR156, detectable H_2_O_2_ level was observed, indicating AR156 pretreatment potentiated plants for an expiated immune response (Figure 2B). At 24 hpi, detectable H_2_O_2_ level was observed in plants inoculated with *B. cinerea*, indicating an initiation of H_2_O_2_-mediated defense response later than 12 hpi but before 24 hpi. In accordance with observation made at 12 hpi, AR156 pretreatment increased the extent of H_2_O_2_ accumulation upon *B. cinerea* infection (Figure 2B). We also compared callose deposition between plants with and without AR156 pretreatment (Figures 2C,D). Similar to H_2_O_2_ accumulation, AR156 alone did not induced any detectable callose deposition. With *B. cinerea* infection, at 12 hpi, callose deposition was detectable, indicating plants were able to perceive the pathogen infection and response efficiently. Moreover, in plants pretreated with AR156, callose deposition was noticeably enhanced, indicating a potentiated defense response due to AR156 pretreatment. In agreement, callose deposition at 24 hpi exhibited similar pattern. Taken together, our results indicate that AR156 primes plants for accelerated and enhanced immune capacity, which is induced only upon pathogen attack and leads to rapidly activated cellular defense responses in systemic tissue.

### AR156-mediated ISR is dependent of the JA/ET signaling pathway and *NPR1*

**Figure 3:**
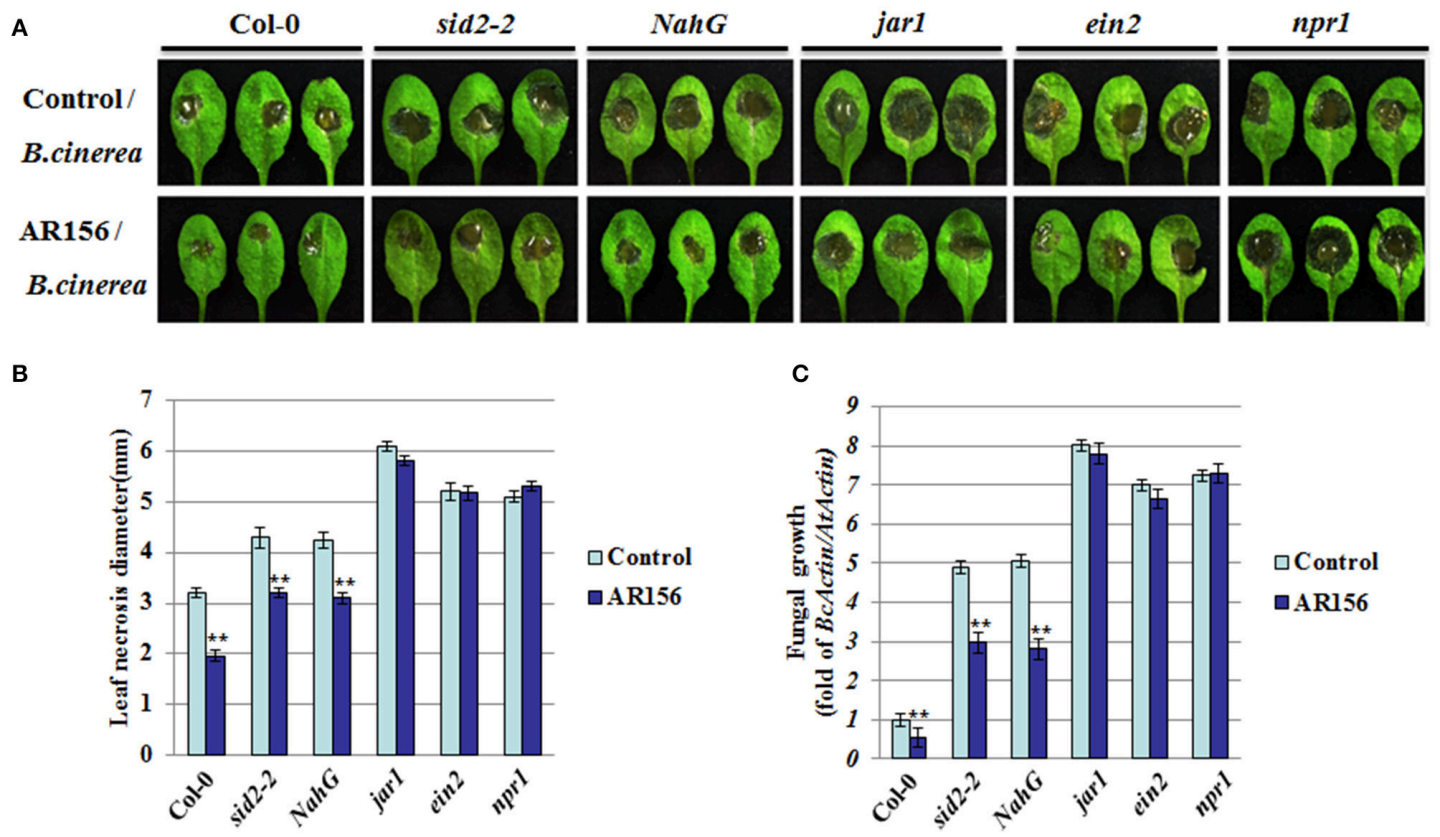
**AR156-mediated ISR through a JA/ET signaling pathway and *NPR1***. Plants pretreatment, *B. cinerea* infection and disease symptom observation are performed as in figure 1. **(A)** Disease symptoms in wild type (Col-0), SA (*sid2-2, NahG*, and *npr1*) and JA/ET (*jar1, ein2*) signaling pathway mutant plants (2 dpi). **(B)** Leaf necrosis development was evaluated at 2 dpi by determining the average necrosis diameter on three leaves per plants for six plants. **(C)** In planta growth of *B. cinerea* in the Col-0, *sid2-2, NahG, jar1, ein2* and *npr1* mutant plants. Measurement of fungal growth was carried out by simultaneous quantification of the transcript levels of *B. cinerea Actin* gene (*BcActin*) and the *Arabidopsis Actin* gene (*AtActin*). Relative fungal growth was determined by ratios of *BcActin/AtActin*. A Student's *t*-test was used to determine significant differences between the AR156-treated sample and the control (^**^P < 0.01). The means values ± *SD* (*n* = 12) from one representative experiment among three independent repeats are shown.

To further elucidate the molecular mechanisms responsible for AR156-triggered ISR, plants defective in different hormone signaling pathways were analyzed. As indicated in Figure 3A, 2 days after pathogen infection, *B. cinerea* caused visible disease symptom on both wild type (Col-0) and *sid2-2, NahG, jar1, ein2, npr1* mutant plants, indicating none of the mutant plants exhibited an automatic and effective immune response against *B. cinerea* infection (Figure 3A, control set). Pretreatment with AR156 led to a reduction in disease symptom on Col-0, *sid2-2* and *NahG* plants, indicting protection mediated by AR156 is still functional in these mutants as effective as in corresponding wild type plants. In contrast, no discernable difference could be observed between the control- and AR156-treated plants on the *jar1, ein2* and *npr1* mutant plants, indicating the AR156-mediated protection was jeopardized in these mutant plants (Figure 3A, AR156 set). In consistence, plants with AR156 pretreatment exhibited a significant (*P* < 0.01) reduction in necrosis size on Col-0, *sid2-2* and *NahG* plants, but not the *jar1, ein2* and *npr1* mutant plants, when compared with control-treated plants (Figure 3B). Fungal growth in each signaling mutant plants were also examined at 2 dpi. In consistence with the disease symptom observed on leaf surface, dramatically reduced fungal growth was detected in Col-0, *sid2-2* and *NahG* plants. In contrast, this protection was abolished in *jar1, ein2* and *npr1* mutant plants (Figure 3C). These results suggested that AR156-mediated ISR functions by activating the JA/ET signaling pathway and is dependent on *NPR1*.

**Figure 4:**
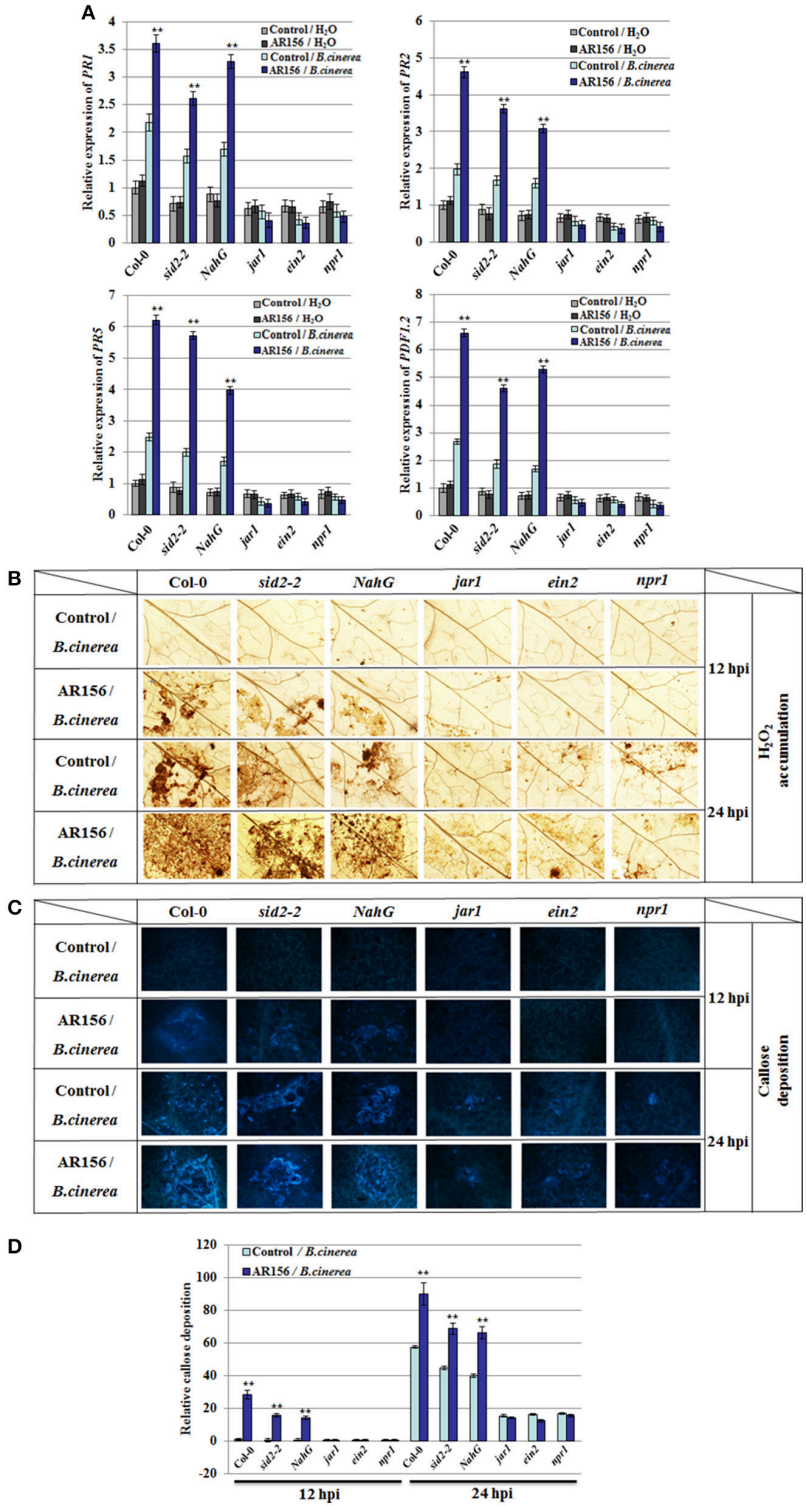
**Altered defense-related gene expression, H_2_O_2_ accumulation and callose deposition in Col-0, *sid2-2*, *NahG*, *jar1*, *ein2*, and *npr1* mutant plants after *B. cinerea* infection**. Four week old plants were infected with spore suspension of *B. cinerea* and leaf samples were taken 12, 24, and 48 hpi, respectively. **(A)** Expression of defense-related genes after *B. cinerea* infection. Expression of defense genes were analyzed by qRT-PCR and normalized with the value of *AtActin*, which is assigned to 1. Data are presented as the means ± *SD* from three independent experiments and different letters above the columns represent statistically significant differences (*p* < 0.01) between Col-0, *sid2-2, NahG, jar1, ein2* and *npr1* mutant plants. *In situ* detection of accumulation of H_2_O_2_ **(B)** and callose deposition **(C, D)** after inoculation with *B. cinerea*. Accumulation of H_2_O_2_ and callose deposition in leaves were detected by DAB staining and aniline blue staining, respectively. Relative callose quantities in droplet inoculated leaves of 4-week-old plants. Callose was quantified from digital microscopy photographs. Shown are mean areas of callose per leaf relative to total leaf area ± *SD* (*n* = 24). A Student's *t*-test was used to determine significant differences between the AR156-treated sample and the control (^**^P < 0.01).

It is known that plant innate immunity is modulated by phytohormone signaling networks. To investigate the signaling pathways employed by AR156-mediated ISR, we inspected the transcription of signaling pathway reporter genes, such as *PR1, PR2, PR5*, and *PDF1.2* by RT-PCR. Transcriptions of *PR1, PR2, PR5*, and *PDF1.2* were strongly induced at 48 hpi in Col-0, *sid2-2* and *NahG* mutant plants pretreated with AR156 and inoculated with *B. cinerea*. Impressively, transcriptions of all these genes were not responsive to *B. cinerea* infection in the *jar1, ein2* and *npr1* mutant plants, perfectly conforming to the defective ISR mediated by AR156 (Figure 4A). Furthermore, cellular defense responses activated by AR156-mediated ISR was investigated in the above-mentioned five signaling mutant and their corresponding wild type *Arabidopsis* plants. H_2_O_2_ accumulation and callose deposition were detectable at 12 hpi in Col-0, *sid2-2* and *NahG* plants with AR156 pre-treatment, but not detectable (or to a much lesser degree) in *jar1, ein2* and *npr1* mutant at the same time point. At 24 hpi, a combination of AR156 pretreatment and *B. cinerea* inoculation led to more H_2_O_2_ accumulation and callose deposition in the leaves of Col-0, *sid2-2* and *NahG* plants, compared with the control-pretreatment group. At the same time point, H_2_O_2_ accumulation and callose deposition were very weak in *jar1, ein2* and *npr1* plants in both control- and AR156-treated plants (Figures 4B–D).

### AR156-induced ISR activates PTI components

**Figure 5:**
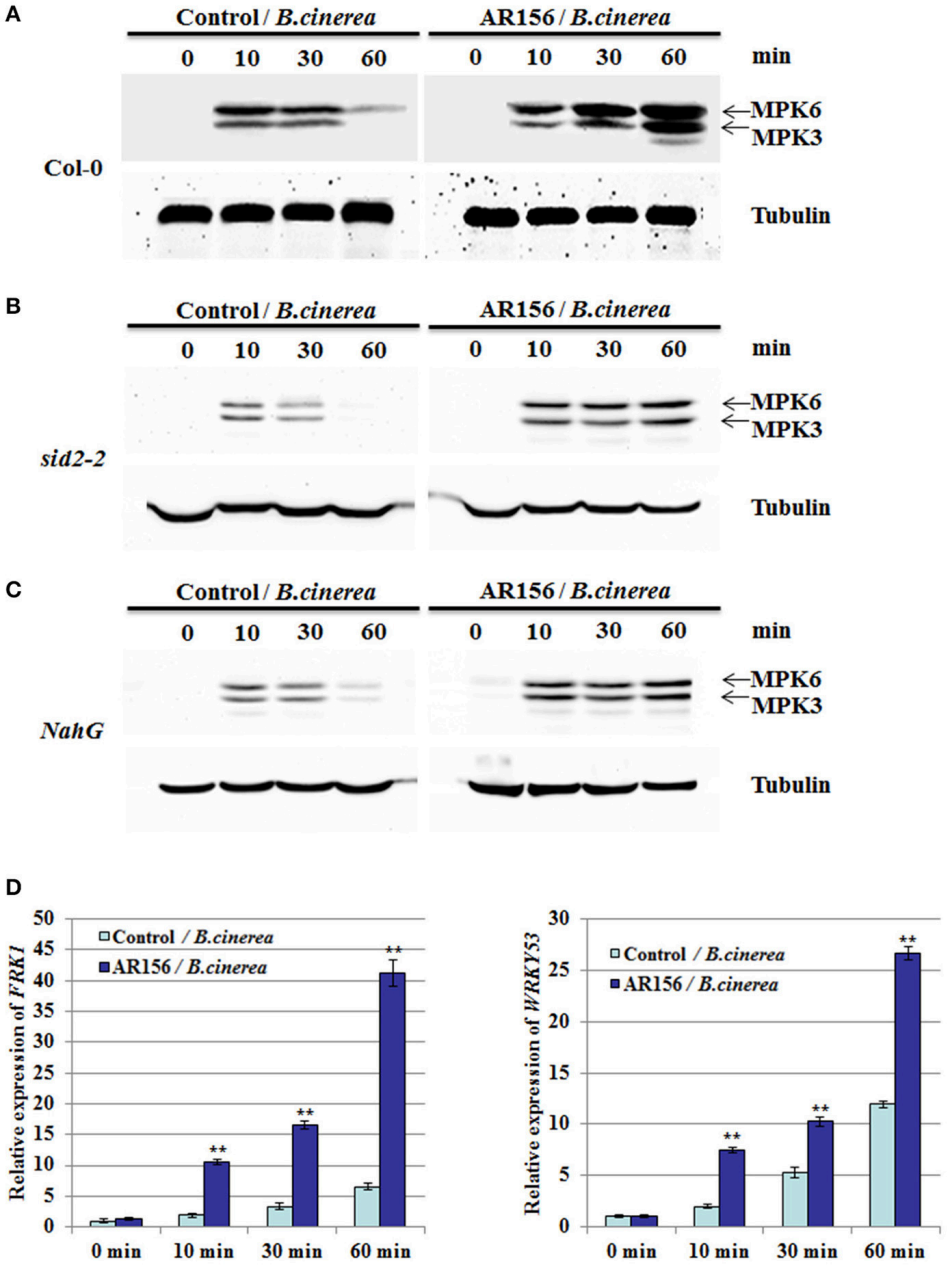
**AR156 activates PTI elements in *Arabidopsis***. *Arabidopsis* Col-0 plants are drench-applied with AR156 at 5 × 10^8^ CFU/ml or 0.85% NaCl (Control). The leaves were infected by *B. cinerea* spore suspension (1 × 10^6^ spores/ml). **(A–C)** Leaves were sampled at 0, 10, 30 and 60 min and MAPKs were detected by Western blot with an anti-Erk antibody (Cell Signaling; #4370S). Tubulin was used as an equal loading control. **(D)** The transcript levels of *FRK1* and *WRKY53* in *Arabidopsis* leaves were analyzed by Real-time RT-PCR. Samples were collected at 0, 10, 30, and 60 min after inoculation with *B. cinerea*. *AtActin* mRNA was used as an internal control. The means values ± *SD* from three independent repeats are shown. A Student's *t*-test was used to determine significant differences between the AR156-treated sample and the control (^**^P < 0.01).

PAMP-triggered immunity (PTI) plays an important role for plant immunity against *B. cinerea* infection, in which activation of MAPKs is one of the earliest sign of PTI. Therefore, activation of MAPKs could be used as a good indicator for activated PTI, as well as defense responses (Eckardt, 2011; Meng and Zhang, 2013; Singh et al., 2013). To further investigate the relationship between AR156-induced ISR and PTI, antibodies specifically recognize MPK3 and MPK6 were used. In plants with control pretreatment, expression of MPK3 and MPK6 were detected 10 min after *B. cinerea* inoculation, which was then gradually decrease through 30 to 60 mpi. In contrast, in plants pretreated with AR156, we observed a sustained and gradually increased activation of MPK3 and MPK6 from 10 to 60 min after *B. cinerea* inoculation, which attained its maximums at 60 min (Figure 5A). Our results suggest that the AR156-mediated ISR is associated with an activated and enduring PTI response. MPK3 and MPK6 activation was also examined in *sid2-2* and *NahG* mutant plants pretreated with AR156. Interestingly, sustained activation of MPK3 and MPK6 from 10 to 60 min was also detected in spite of the defect on *SID2* (Figure 5B) or over-expression of *NahG* (Figure 5C). Our results indicate that MPK3 and MPK6 induction is SA-dependent. This may explain why AR156-mediated ISR is SA-independent.

To further support our hypothesis, we examined other PTI marker gene, such as the *flg22-induced receptor-like kinase 1* (*FRK1*) and *WRKY53* (Asai et al., 2002; Singh et al., 2012). qRT-PCR analysis showed that *FRK1* and *WRKY53* were activated and remained active by *B. cinerea* alone from 10 to 60 min. However, in AR156-pretreated plants, both the expression level was significantly increased after *B. cinerea* infection, when compared to the control-pretreated plants (Figure 5D). These results confirmed that there is a close association between AR156-mediated ISR and a rapid and sustained activation of PTI.

## Discussion

Induced systemic resistance (ISR) has been recognized as an effective biological control agent that could induce plant defense against a broad range of pathogens. Our precious studies demonstrated that *B. cereus* AR156 is a plant growth–promoting rhizobacterium that can induce resistance against *Pst* DC3000 on *Arabidopsis* and tomato (Niu et al., 2011, 2012). In particular, our study in *Arabidopsis* showed that the AR156-mediated ISR against the biotrophic pathogen *Pst* DC3000 is dependent on SA and *NPR1*, but not the ET/JA signaling pathway. In the current study, we demonstrated that the AR156-mediated ISR is also effective against the necrotrophic pathogen *B. cinerea*, and this protection is mediated by the ET/JA-, but not the SA-signaling pathway. However, we did identified *NPR1* as an indispensable component of this specific ISR, despite the fact that other SA signaling pathway components were not involved. Therefore, our results indicate that the AR156-mediated ISR is effective against both bio- and necro-trophic pathogens, which make it a good candidate for broad-spectrum biological control agent. Meanwhile, our results also indicate that *NPR1* could potentially function independent of the SA signaling pathway.

In recent years, many studies have associated PGPRs with improving plant health by enhancing defense against a broad range of pathogens (Pieterse et al., 2014; Rahman et al., 2014; Ma et al., 2016). *B. cereus AR156* is a plant growth–promoting rhizobacterium that induces resistance against *Pst* DC3000 on *Arabidopsis* and tomato (Niu et al., 2011, 2012). The aim in this study was to explore the potential role of AR156 in eliciting ISR in *Arabidopsis* against *B. cinerea* infection. Roots application of AR156 significantly reduced necrosis diameter and inhibited fungal growth on the leaves of *Arabidopsis* plants (Figure 1). Since AR156 only colonized the roots but *B. cinerea* was a foliar applied, the lack of direct contact between these two parties indicates that it is ISR, instead of a direct limitation of pathogen, leads to the observed resistance to *B. cinerea* infection.

Under primed condition, the induction of defense-related *PR* genes following pathogen challenge has been reported in several plant-pathogen interaction (Ahn et al., 2007; Niu et al., 2011). This is also true in ISR against several hemibiotrophic and necrotrophic pathogens, such as the *Harpophora oryzae*-primed defense genes in the rice–*Magnaporthe. oryzae* interaction (Su et al., 2013) and *Bacillus subtilis*-induced *PR* genes in tomato challenged with *Erwinia carotovora* subsp. *carotovora* (Chandrasekaran and Chun, 2016). In this study, we found that PR1 protein expression was stronger in plants with AR156 pretreatment and *B. cinerea* infection than that in plants with pathogen infection only (Figure 2A). This is in consistence with reported results (Su et al., 2013; Chandrasekaran and Chun, 2016) and suggests that the induced PR expression level also contributes to defense against hemibiotrophic and necrotrophic pathogens. Rapid production of cellular defense responses in plant cells, such as quick H_2_O_2_ accumulation and callose deposition (Conrath et al., 2002), has been recognized as hallmark events induced by ISR-triggering bacteria (Ahn et al., 2007; Rahman et al., 2014). H_2_O_2_ and callose accumulation play important roles in plants response to *B. cinerea* infection (Mengiste, 2012; Schwessinger and Ronald, 2012). We found that AR156-pretreated plants accumulated higher H_2_O_2_ and callose levels than control-treated plants following *B. cinerea* infection (Figures 2B–D), suggesting that AR156 primes plants for accelerated and enhanced disease resistance capacity by activating cellular defense responses in systemic tissue.

Therefore, it interesting to seek how the defense signal is transmitted to remote tissues where the pathogen threaten has not reach yet. We investigated the involvement of SA and JA/ET signaling pathway components, which were previously demonstrated to be involved in the AR156-induced defense responses (Niu et al., 2011, 2012, 2016). AR156-induced ISR was abolished in *jar1* and *ein2* mutants, suggesting that the defense response is induced by AR156 through JA/ET-signaling pathways. This observation is in consistence with that the JA/ET-signaling pathways are more effective against infections by necrotrophic pathogens. Components involved in the SA signaling pathway were also examined. In our study, both *sid2-2* and *NahG* plants showed comparable disease symptoms to wild type plants, and similar necrosis diameter and fungal growth in plants pretreated with AR156. *SID2* is one of the genes involved in SA synthesis, defect of which leads to reduced cellular SA accumulation. *NahG* plants are transgenic *Arabidopsis* expressing an SA hydroxylase (*NahG*) that degrades SA to catechol. Unaffected ISR in these two independent SA defective plants indicates that the AR156-mediated ISR against *B. cinerea* is independent of cellular SA level, and pretty much neither of the SA signaling pathway. ISR in *NahG* transgenic plants and defective of ISR by *jar1, ein2* and *npr1* have been reported in *Arabidopsis* previously (Pieterse et al., 1996; Ryu et al., 2003). However, there is a difference between the previous findings and ours that in their experiment, rhizobacterium treatment was not associated with induction of *PR* gene (van Wees et al., 1999; Verhagen et al., 2004), whereas augmented PR1 protein level was observed in *Arabidopsis* pretreated with AR156 and then infected with *B. cinerea* (Figure 2A).

Interestingly, we previously demonstrated that in AR156-mediated ISR against the biotrophic *Pst* DC3000, both the SA- and JA/ET-signaling pathways were simultaneously activated (Niu et al., 2011). In contrast, when we analyze the AR156-mediated ISR against the necrotrophic *B. cinerea* in this study, we concluded that this type of ISR was accompanied by the activation of the JA/ET-signaling pathways, but not the SA signaling pathway. It is generally accepted that biotrophic pathogens, which acquire nutrient supply from live host cells, are more vulnerable to defense through SA-signaling pathway; whereas necrotrophic pathogens, which benefit from host cell death, are better restrained by a JA/ET-dependent defense (Grant and Lamb, 2006). So we speculated that the favorable signal transduction pathway promoted during ISR not only depends on the ISR-inducing strains and the host plants (Pieterse et al., 2002; Choudhary and Johri, 2009; Shoresh et al., 2010), but also on the pathogens the ISR apply to.

Intriguingly, AR156-induced ISR was noticeably jeopardized in the *npr1* mutant, the gene of which encodes a redox-sensitive transcriptional regulator of SA-dependent responses. *NPR1* also is a mediator of SA-JA cross talk, and a regulator of SAR and ISR (Pieterse et al., 2014). Upon activation by SA, NPR1 acts as a transcriptional coactivator of a large set of *PR* genes as we observed in our study. This clearly laid a discrepancy between the induction of SA-dependent *PRs* and the independency on SA synthesis and cellular content, which may suggest a SA-unrelated function of *NPR1*. Indeed, *NPR1* was shown to be required for the SA-independent but JA/ET-dependent ISR triggered by *P. fluorescens* WCS417r (Pieterse et al., 1998). More and more evidence point to a cytosolic function of *NPR1* in JA/ET signaling and ISR (Spoel et al., 2003; Ramirez et al., 2010; Pieterse et al., 2012). It is worthy to note that *NPR1* are highly expressed in *Arabidopsis* roots (Iyer-Pascuzzi et al., 2011), which may imply a potential role in regulating root-associated immune responses including ISR.

Our results also showed that, in AR156-primed *Arabidopsis*, pathogen infection triggered expression of defense-related genes, and enhanced hydrogen peroxide accumulation and callose deposition (Figure 2). However, we found that induced expression of *PR1, PR2* and *PR5* was also observed in *sid2-2* and *NahG* mutants, which are defective for SA accumulation. This is a surprise to us because *PR1, PR2*, and *PR5* were generally recognized as markers of salicylic acid-dependent disease responses, which should be non-responsive in SA-deficient mutants (Vlot et al., 2009). Our results suggest that in the AR156-mediated ISR against *B. cinerea, PR1, PR2* and *PR5* were induced through a SA-independent signaling pathway. This is supported by a prior study in which constant *MPK3* and/or *MPK6* activation causes *PR1* induction independent of SA (Tsuda et al., 2013). Taken together, *PRs* gene activation in *sid2*-2 and *NahG* by AR156 pretreatment and *B. cinerea* infection could be a consequence of activated JA/ET signal pathways and induced MAPKs cascade.

Previous studies already indicate the importance of MAPK signaling in plant defense against infections (Asai et al., 2002; Zipfel et al., 2004). In this study, MAPK activation was detected at 10 min and decreased at 60 min in the leaves of plants only inoculated with *B. cinerea*, but this induction initiated at about the same time but remained very strong at 60 min in those treated with AR156 and inoculated with *B. cinerea* (Figure 4A). This indicated that AR156-pretreatment induced stronger MAPK activation than plants without pretreatment. MPK3 and MPK6 are positive regulators of plant defense responses controlling ET (Tena et al., 2011; Meng and Zhang, 2013) and JA biosynthesis (Schweighofer and Meskiene, 2008). MPK3 and MPK6 are essential for plant defense against *B. cinerea* (Ren et al., 2008; Han et al., 2010; Galletti et al., 2011; Mendez-Bravo et al., 2011). This is consistent with our finding that AR156-induced ISR against *B. cinerea* is mediated by JA/ET-signaling pathways. qRT-PCR analysis of the MAMP-specific early-defense marker genes, such as *FRK1* and *WRKY53* showed that MAMP-mediated defense responses occur rapidly after treatment with AR156 and inoculation with pathogen (Figure 5D), implying that AR156 induces SAR through the activation PTI response.

**Figure 6:**
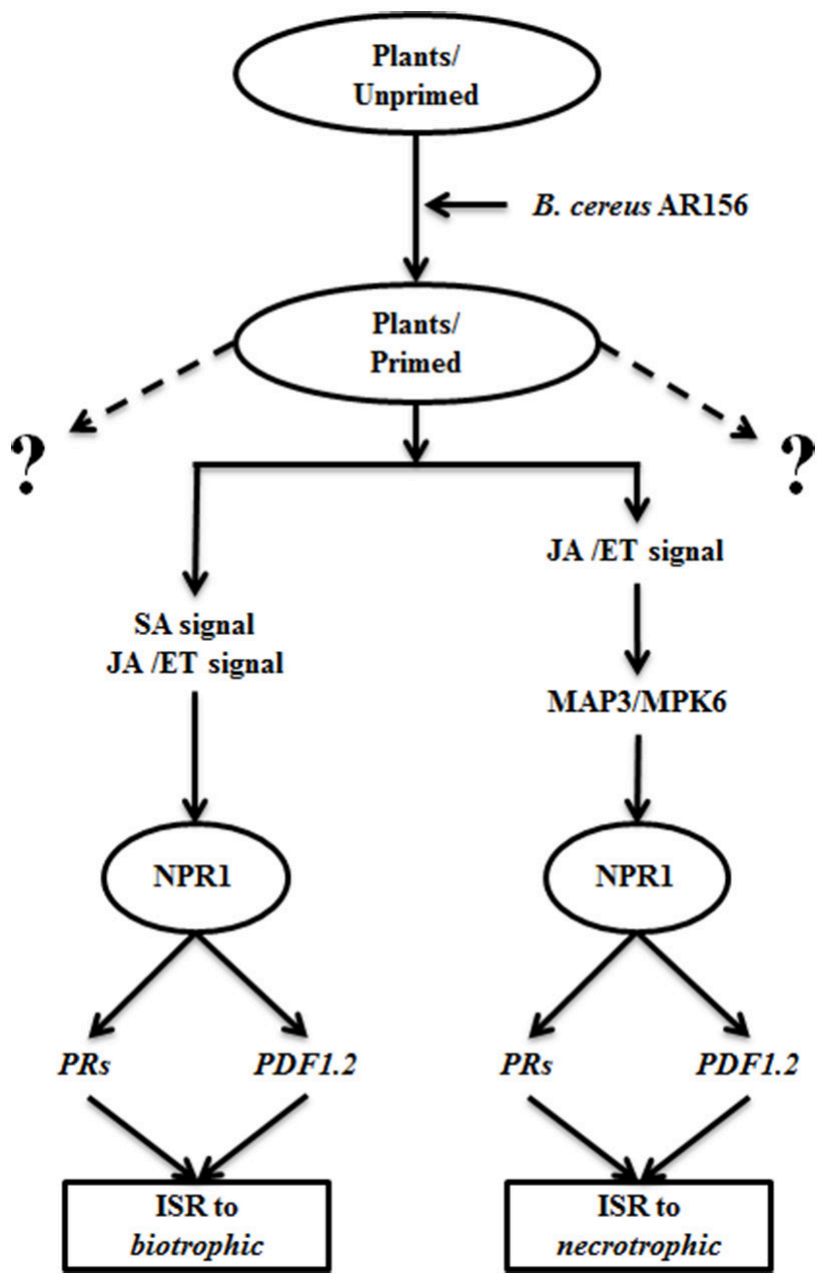
**A proposed model of AR156-mediated ISR against biotrophic and necrotrophic pathogens**. When plants are pretreated with AR156, an ISR omnipotent to pathogens with both biotrophic and necrotrophic life styles is induced. When plants in a potentiated immune status is infected by a biotrophic pathogen, such as *Pst* DC3000, both SA and JA/ET signaling pathways are activated. Through a mechanism dependent on *NPR1*, downstream defense-related genes, such as *PR1, PR2, PR5*, and *PDF1.2* are expressed, and cellular defense responses, such as H_2_O_2_ accumulation, callose deposition are activated; when plants are challenged with necrotrophic pathogens, such as *B. cinerea*, only the JA/ET signaling pathways is activated. The necrotrophic-effective ISR is also dependent on *NPR1*. Dashed lings: protective function to other unidentified elicitors.

In our previous study, the AR156-mediated ISR could efficiently protect plants against infections by biotrophic pathogen, such as *Pst* DC3000. This ISR simultaneously activate the SA- and the JA/ET-dependent signaling pathways, as evident by the induced expression of *PR1, PR2, PR5*, and *PDF1.2* (Niu et al., 2011). In this study, we demonstrated that AR156 was also effective in protecting plant from infection by necrotrophic pathogens, such as *B. cinerea*. However, in this case, the JA/ET signaling pathways but not the SA signaling pathway is involved. In both cases the induced ISR is associated with similar defense responses, such as activated cellular defense responses, such as H_2_O_2_ accumulation, callose deposition, and expression of some defense related genes. Therefore, we propose a model that AR156-mediated ISR is effective against infection by pathogens with different life cycles. In this model, when plants are pretreated with AR156, ISR is activated by equipping plants with a potentiated immune status that is omnipotent to pathogens with both biotrophic and necrotrophic life styles. When plants in potentiated status are infected by a biotrophic pathogen, such as *Pst* DC3000, both SA and JA/ET signaling pathways are activated. Through a mechanism dependent on *NPR1*, downstream defense-related genes, such as *PR1, PR2, PR5*, and *PDF1.2* are expressed, and cellular defense responses, such as H_2_O_2_ accumulation, callose deposition are activated; when plants are challenged with necrotrophic pathogens, such as *B. cinerea*, only the JA/ET signaling pathways is activated. Once again through a *NPR1*-dependent mechanism, downstream defense responses are activated, leading to increase resistance (Figure 6). However, whether the role of *NPR1* is conserved between the SA and JA/ET signaling pathways is unclear to us. Further study is needed to clarify the versatile function of *NPR1* in AR156-mediated ISR, which is of great significance in promoting the application of AR156 in crops protection.

## Author contributions

DN and HZ designed the study. PN, XL, and SW performed the experiments. All authors analyzed the data. DN and HZ wrote the manuscript. All authors contributed to the research and approved the final version of the manuscript.

### Conflict of interest statement

The authors declare that the research was conducted in the absence of any commercial or financial relationships that could be construed as a potential conflict of interest.
