# Supplementary material for: Induced Systemic Resistance against Botrytis cinerea by Bacillus cereus AR156 through a JA/ET- and NPR1-Dependent Signaling Pathway and Activates PAMP-Triggered Immunity in Arabidopsis
Source: Front Plant Sci. 2017 Feb 28;8:238. doi: 10.3389/fpls.2017.00238 (PMC5329000; doi:10.3389/fpls.2017.00238)
Supplement: Supplementary file 1 [file Table1.DOC]

**Table S1. Primer used for gene expression analysis in this study.**

| **Gene name** | **Gene ID** | **Forward primer (5’-…-3’)** | **Reverse primer (5’-…-3’)** |
| --- | --- | --- | --- |
| ***PR1*** | **At2g14610** | CTCGGAGCTACGCAGAACAA | TTCTCGCTAACCCACATGTTCA |
| ***PR2*** | **At3g57260** | CGGTACATCAACGTTGGAA | GCGTAGTCTAGATGGATGTT |
| ***PR5*** | **At1g75040** | ATGGCAAATATCTCCAGTATTCACA | ATGTCGGGGCAAGCCGCGTTGAGG |
| ***PDF1.2*** | **At5g4420** | AGTTGTGCGAGAAGCCAAGT | GTTGCATGATCCATGTTTGG |
| ***Actin*** | **At1g01130** | GGCGATGAAGCTCAATCCAAACG | GGTCACGACCAGCAAGATCAAGACG |
| ***FRK1*** | **At2g19190** | GCCAACGGAGACATTAGAG | CCATAACGACCTGACTCATC |
| ***WRKY53*** | **At4g23810** | CACCAGAGTCAAACCAGCCATTAC | CTTTACCATCATCAAGCCCATCGG |
